# Donor Screening Failure for *Strongyloides stercoralis* in Solid Organ Transplantation

**DOI:** 10.3201/eid3201.251483

**Published:** 2026-01

**Authors:** Rocio Kohan, Helena Gil-Campesino, Inés O. García Rodríguez, Magdalena Lara

**Affiliations:** Hospital Universitario de Canarias, San Cristóbal de La Laguna, Canary Islands, Spain (R. Kohan, I.O. García Rodríguez); Hospital Universitario Nuestra Señora de Candelaria, Santa Cruz de Tenerife, Canary Islands, Spain (H. Gil-Campesino, M. Lara).

**Keywords:** *Strongyloides stercoralis*, solid organ transplant, screening, serology, hyperinfection, hypereosinophilia, Loeffler’s syndrome, parasites, parasitic infections, Canary Islands, Spain

## Abstract

We report 2 cases of donor-derived *Strongyloides stercoralis* infection in renal transplant recipients. Despite initial negative serologic testing in donor samples, retrospective testing confirmed transmission. This report underscores the limitations of serologic screening, the need for targeted protocols in endemic-risk populations, and the importance of close posttransplant surveillance.

*Strongyloides stercoralis* can persist for decades in humans (*1*). In immunocompromised patients, such as transplant recipients, *S. stercoralis* can cause disseminated infection or hyperinfection syndrome (*2*). Mortality exceeds 60% in immunosuppressed persons (*3*), reaching 87% if treatment is not initiated (*4*). We report 2 cases of strongyloidiasis in renal transplant recipients who shared the same donor.

The donor was a 72-year-old man who was born in Ghana and resided in Tenerife, Canary Islands, Spain, for 20 years. He died from subarachnoid hemorrhage after a traumatic brain injury. He had no known immunosuppressive condition, and his eosinophil count was unremarkable. Following National Transplant Organization guidelines on the selection criteria of donors in relation to infectious diseases (*5*), routine donor serologic screening was conducted. Testing for HIV, human T-lymphotropic virus 1 and 2, hepatitis C and B virus, syphilis, cytomegalovirus, Epstein-Barr virus, herpes simplex virus 1 and 2, and toxoplasmosis revealed no noteworthy findings except positive results for cytomegalovirus IgG and hepatitis B core antibody (hepatitis B surface antigen was negative). Because of the donor’s geographic origin, Mantoux testing and testing for antibodies against *Coccidioides immitis*, *Histoplasma capsulatum*, *Plasmodium* spp., and *S. stercoralis* were conducted; all results were negative. The additional testing was conducted at an external reference laboratory (Reference Laboratory S.A., Barcelona, Spain), where *Strongyloides* serology was performed by using a crude-antigen enzyme-linked immunosorbent assay (SciMedx Corporation, https://www.scimedx.com), yielding an index value of <0.1 (>1 is considered positive). One kidney from the donor was transplanted into each of 2 recipients.

Recipient A was a 65-year-old man with diabetic nephropathy who was on continuous ambulatory peritoneal dialysis for 11 months before transplant. No complications were reported during the immediate posttransplant period. Approximately 2 months after transplant, a cough with sputum production developed; the patient was initially treated on an outpatient basis with oral amoxicillin/clavulanic acid (500 mg/8 h for 7 days). He then sought care at the local emergency department with severe epigastric pain and heartburn, asthenia, tendency toward hypotension, and persistence of mild respiratory symptoms. A computed tomography scan revealed gastric distension, mild small-bowel dilatation and thickening, minimal pelvic free fluid, and bilateral pleural and pericardial effusion. Laboratory findings included C-reactive protein of 114 mg/L (reference range <5 mg/L), increased leukocytes of 23 × 10^9^ cells/L (reference 4–11 × 10^9^ cells/L ), serum IgE 1,664.9 IU/mL (reference <100 IU/mL), and marked hypereosinophilia, rising from 3.2 × 10^9^ cells/L (reference <0.5× 10^9^ cells/L) at admission to 18.7 × 10^9^ cells/L on day 6 of hospitalization (Figure 1). We learned the patient had previously traveled by caravan across Spain’s Mediterranean coast, an area described as endemic for strongyloidiasis (*6*). Formalin ethyl-acetate sedimentation concentration with subsequent microscopic examination of stool samples revealed *S. stercoralis* L1 rhabditiform larvae (Figure 2). Because of the donor’s negative serology, we suspected endogenous reactivation. The patient received oral ivermectin (200 μg/kg/d for 25 days) and albendazole (400 mg 2×/d for 15 days), with progressive clinical improvement. Once the patient reached normalization of eosinophilia and preservation of graft function, he was discharged.

**Figure 1:**
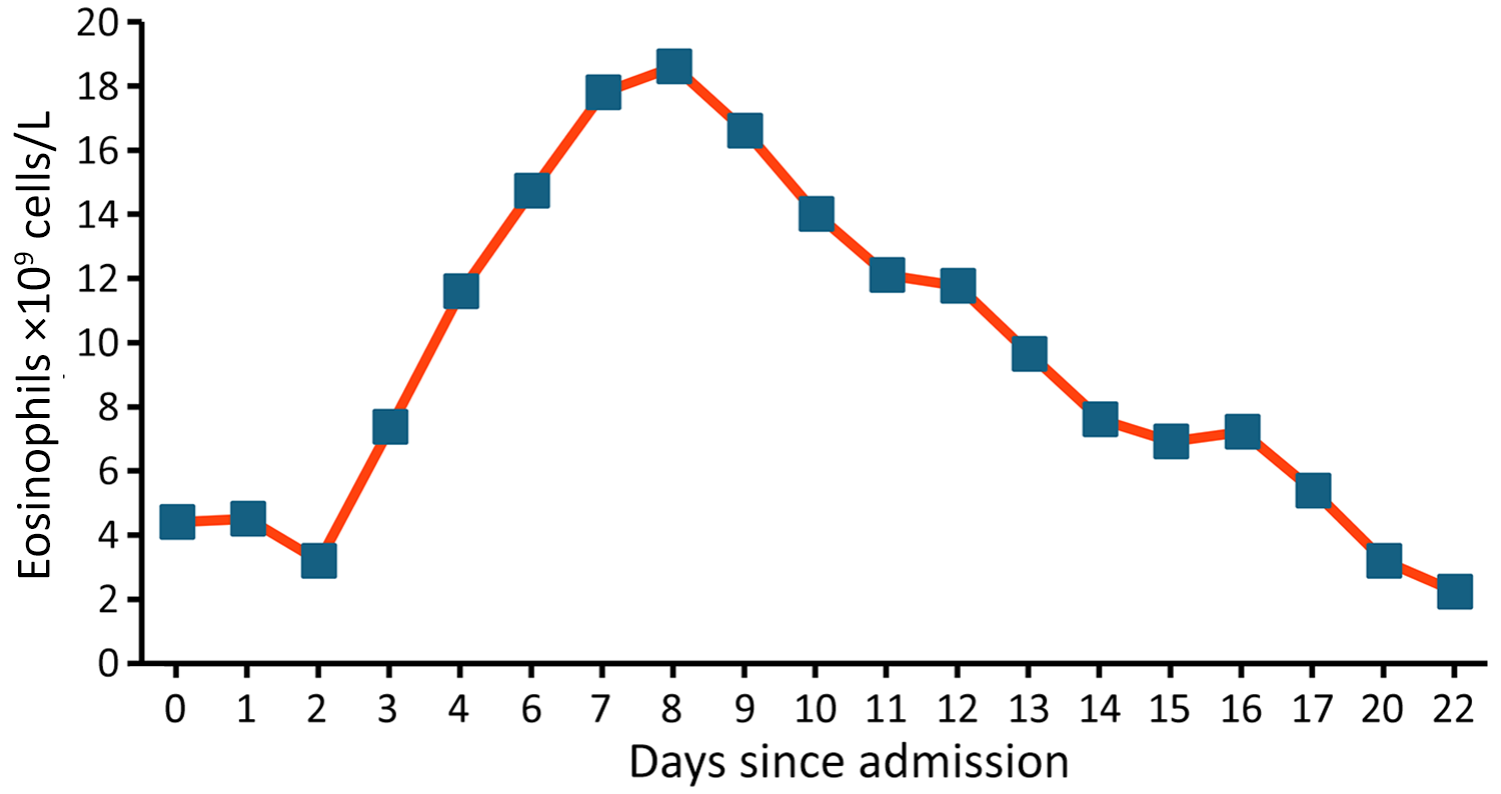
Eosinophil count evolution in recipient A during admission in a study of donor screening failure for *Strongyloides stercoralis* in solid organ transplantation.

**Figure 2:**
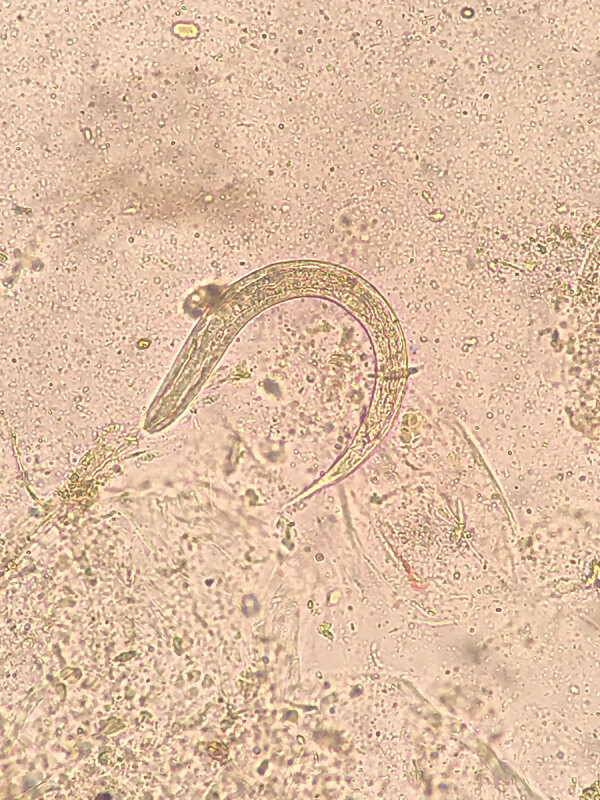
*Strongyloides stercoralis* L1 rhabditiform larva found in stool sample from recipient A in a study of donor screening failure for *Strongyloides stercoralis* in solid organ transplantation.

Recipient B was a 74-year-old man with diabetic nephropathy who was on hemodialysis for 20 months before transplantation; no complications were reported during the immediate posttransplant period. He had no known risk factors for *Strongyloides* infection. After the confirmed diagnosis in recipient A, we conducted targeted screening, revealing *S. stercoralis* L1 rhabditiform larvae in stool samples examined by direct microscopy by using the formalin ethyl-acetate sedimentation concentration technique. He did not show peripheral eosinophilia at any time and remained asymptomatic with unremarkable laboratory values throughout follow-up. We treated him with oral ivermectin (200 μg/kg/d for 2 days) and repeated treatment 2 weeks later. Both patients tested negative in follow-up stool examinations performed by using the same concentration and microscopy methods.

Because of the suspicion of a common transmission source, stored donor serum was sent for repeat *Strongyloides* serologic testing by using third-stage larvae antigen enzyme-linked immunosorbent assay for *Strongyloides* IgG (DRG Instrument Gmbh, https://www.drg-diagnostics.de). The sample tested positive with an index value of 1.61 (>1.1 is considered positive), confirming donor-derived transmission of the parasite.

This event highlights several clinically relevant considerations. First, the sensitivity of serologic assays varies widely (*7*,*8*); thus, active infection cannot be ruled out solely on the basis of a negative result in epidemiologically at-risk patients. This limitation is even more relevant for immunosuppressed patients, in whom serologic testing might yield false-negative results. Therefore, additional diagnostic methods, such as PCR, stool concentration techniques, or agar plate culture, should be considered to reliably exclude infection. Second, it underscores the need to reinforce targeted screening protocols in both donors and recipients with origin or prolonged residence in endemic areas, including national regions (*9*), which are often overlooked. Close monitoring of recipients from such donors remains essential, and the appearance of any new symptoms, such as mild respiratory symptoms seen in recipient A, should raise concern, even with negative donor serology, as in this case it might corresponded to Löffler syndrome. Finally, this report highlights the usefulness of storing serum from deceased donors for retrospective confirmation.
